# Supplementary material for: Exceptional points, lasing, and coherent perfect absorption in Floquet scattering systems
Source: Light Sci Appl. 2026 Sep 9;15:375. doi: 10.1038/s41377-026-02380-9 (PMC13558774; doi:10.1038/s41377-026-02380-9)
Supplement: Supplementary file 1 [file 41377_2026_2380_MOESM1_ESM.pdf]

Supplementary Information for Exceptional Points, Lasing, and Coherent Perfect Absorption in Floquet Scattering Systems

David Globosits,[1,*] Puneet Garg,[2,*] Jakob Hüpfl,[1] Adrià Canós Valero,[3,4] Thomas Weiss,[3] Carsten Rockstuhl,[2,5] and Stefan Rotter[1]

[1] *Institute for Theoretical Physics, Vienna University of Technology (TU Wien), 1040 Vienna, Austria*
[2] *Institute of Theoretical Solid State Physics, Karlsruhe Institute of Technology, 76131 Karlsruhe, Germany*
[3] *Institute of Physics, University of Graz, and NAWI Graz, 8010 Graz, Austria*
[4] *Riga Technical University, Institute of Telecommunications, 1048 Riga, Latvia*
[5] *Institute of Nanotechnology, Karlsruhe Institute of Technology, 76131 Karlsruhe, Germany*

S1. CONNECTION OF S_{F} TO BAND STRUCTURE

We mention here that the Floquet scattering matrix also provides access to the band structure of the underlying scattering systems [1], which we interpret here as the spectral location their resonant states in the parameter space. The resonant states of a photonic structure are the self-standing modes that exist without any incident excitation. In general, for planar geometries, the following relation holds

$$S_{\mathrm{F}}^{-1}(\omega, \mathbf{k}_{\|}) \left|c^{\mathrm{out}}\right\rangle = \left|c^{\mathrm{in}}\right\rangle \tag{S1}$$

For such a resonant state, we require $\left|c^{\mathrm{out}}\right\rangle \neq 0$ given $\left|c^{\mathrm{in}}\right\rangle = 0$. Such a condition holds if

$$\sigma_{\min}\{S_{\mathrm{F}}^{-1}(\omega, \mathbf{k}_{\|})\} \to 0 \tag{S2}$$

where $\sigma_{\min}$ represents the minimum singular value. Those values of ω and $\mathbf{k}_{\|}$ that satisfy Eq. (S2) constitute the location of an eigenmode in the parameter space. Note that ω is in general complex due to the radiation and material losses of finite scattering structures.

S2. FURTHER DETAILS ON THE CPA AND LASING STATES

Here, we provide further details on the spatial and spectral distribution of the eigenstates of the Floquet scattering matrix for the periodically time-varying slab at normal incidence. Furthermore, we analyze the property of pseudophoton and photon conservation for eigenstates of S_{F}.

In Fig. S1a, we depict the evolution of the four eigenvalues of the Floquet scattering matrix expressed in a photon-flux normalized basis that undergo a symmetry breaking transition. It contains the same data as Fig. 1d of the main text. Here, we also choose the same parameters for the slab as in the main text. We select four eigenvalues at different modulation amplitudes indicated by the black crosses (two in the unbroken regime and two in the broken regime) and investigate the associated eigenstates in detail.

In Fig. S1b, we depict the corresponding eigenstates to these four eigenvalues. In panel (i), we plot the distribution of the entries of the photon-flux normalized eigenstate corresponding to a unimodular eigenvalue and the associated outgoing state. Specifically, we plot the entries corresponding to the left asymptotic region, i.e., $|c_{\mathrm{l},n}^{\mathrm{in}}|^2$ (orange) and $|c_{\mathrm{l},n}^{\mathrm{out}}|^2$ (green). Due to the spatial symmetries of the scattering system, the distribution of the entries of the eigenstate for the right asymptotic region is identical to the left one. Therefore, we only plot the distribution corresponding to the left asymptotic region. In panel (i), we observe that the distributions of the incoming and outgoing coefficients are the same, as expected for an eigenstate to a unimodular eigenvalue λ_{ν_0} of the Floquet scattering matrix:

$$\mathbf{c}_{\nu_0}^{\mathrm{out}} = S_{\mathrm{F}} \mathbf{c}_{\nu_0}^{\mathrm{in}} = \lambda_{\nu_0} \mathbf{c}_{\nu_0}^{\mathrm{in}} \tag{S3a}$$

$$\Rightarrow \left|\left(\mathbf{c}_{\nu_0}^{\mathrm{out}}\right)_n\right|^2 = |\lambda_{\nu_0}|^2 \left|\left(\mathbf{c}_{\nu_0}^{\mathrm{in}}\right)_n\right|^2 = \left|\left(\mathbf{c}_{\nu_0}^{\mathrm{in}}\right)_n\right|^2 \tag{S3b}$$

Furthermore, we see that the eigenvector consists of both positive- and negative-frequency components. Interestingly, for the specific state depicted in panel (i) of Fig. S1b the corresponding incoming photon flux associated with the positive-frequency channels given by $\sum_{n\geq 0} \left|\left(\mathbf{c}_{\nu_0}^{\mathrm{in}}\right)_n\right|^2$ is larger than the corresponding negative-frequency photon flux $\sum_{n<0} \left|\left(\mathbf{c}_{\nu_0}^{\mathrm{out}}\right)_n\right|^2$ (orange solid lines). In accordance with Eq. (S3b), however, we see that the outgoing photon flux (green dashed lines) is the same as the incoming one. Thus, each eigenstate in the unbroken regime individually conserves the number of photons during scattering. Furthermore, the incoming and outgoing photon fluxes associated with the positive and negative frequencies are the same, confirming that the number of pseudophotons is conserved for this state, such that

$$\sum_n \mathrm{sign}(\omega_n) \left|\left(\mathbf{c}_{\nu_0}^{\mathrm{out}}\right)_n\right|^2 = \sum_n \mathrm{sign}(\omega_n) \left|\left(\mathbf{c}_{\nu_0}^{\mathrm{in}}\right)_n\right|^2 \tag{S4}$$

* These authors contributed equally: David Globosits, Puneet Garg

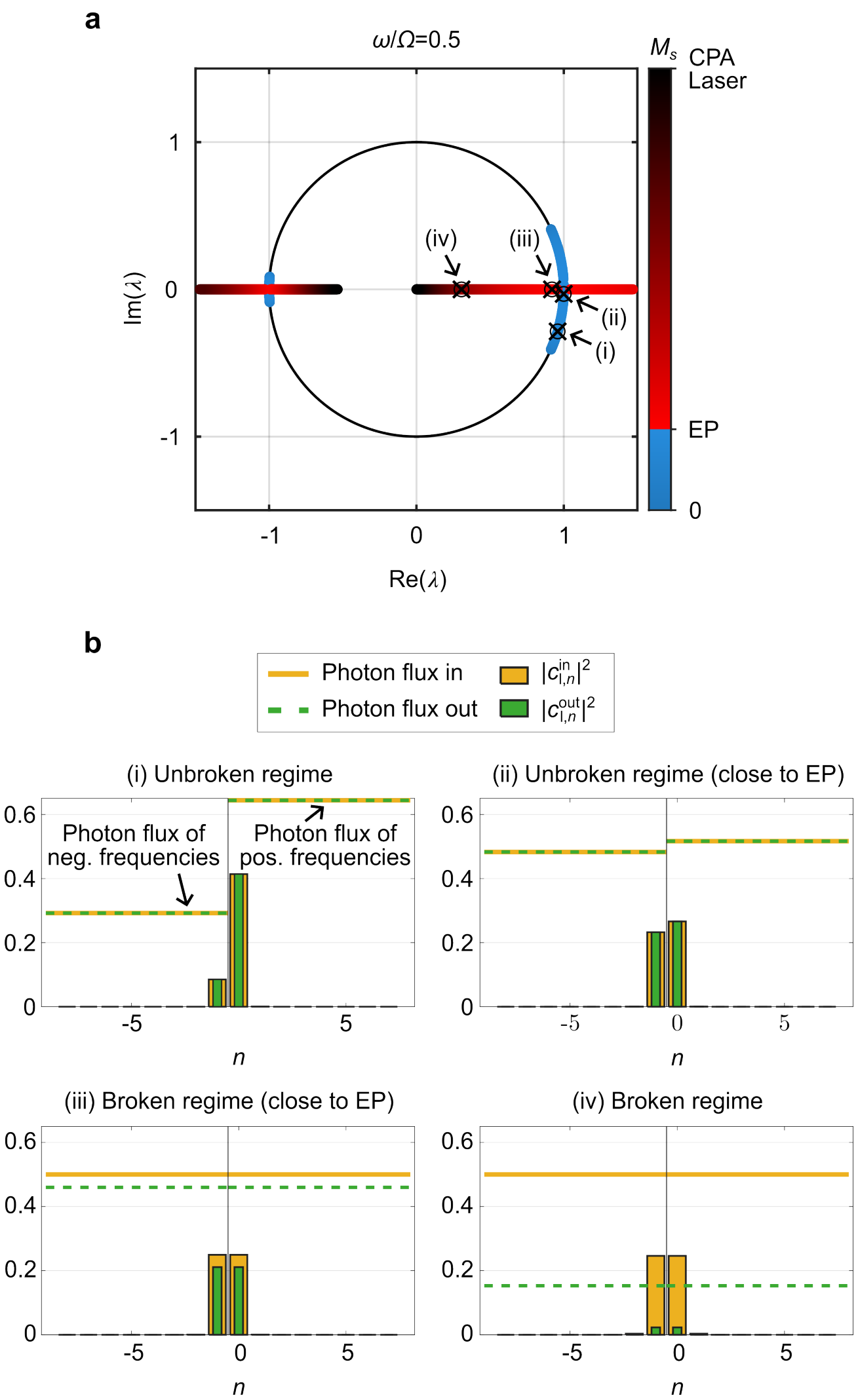

FIG. S1. **a** We track four eigenvalues of S_{F} as a function of the modulation strength. For small M_{s} all eigenvalues are unimodular and thus lie on the unit circle (blue). At a critical modulation strength, EPs are formed, where two respective eigenvalues coincide. By further increasing M_{s}, these two eigenvalues leave the unit circle in a pairwise fashion (red). **b** We select four eigenvalues indicated by black crosses in **a** and plot the distribution of the components of the corresponding eigenstates of S_{F} (orange bars). Furthermore, we depict the distribution of the associated output light field (green bars). The orange solid line represents the respective photon flux of the incoming state and the green dashed line represents the photon flux of the outgoing state. In panels (i) and (ii) we choose states from the unbroken symmetry regime, while panels (iii) and (iv) show states from the broken regime.

In panel (ii), we depict an eigenstate corresponding to an eigenvalue that is close to the EP but still in the unbroken regime. We again observe that the incoming and outgoing photon flux is the same, indicating that this state conserves the number of photons (and also the number of pseudophotons). We highlight that being close to the EP, this state has nearly balanced photon flux associated with the positive- and negative-frequency components.

In panel (iii), we show an eigenstate in the broken regime still being close to the EP. We see that the distribution of the components of the outgoing state is a rescaled (by a factor $\left|\lambda_{\nu_-}\right| < 1$) version of the distribution of the incoming eigenstate. We can understand this by again considering the eigenvalue relation:

$$\mathbf{c}_{\nu_-}^{\mathrm{out}} = S_{\mathrm{F}}\mathbf{c}_{\nu_-}^{\mathrm{in}} = \lambda_{\nu_-}\mathbf{c}_{\nu_-}^{\mathrm{in}} \tag{S5a}$$

$$\Rightarrow \left|\left(\mathbf{c}_{\nu_0}^{\mathrm{out}}\right)_n\right|^2 = \left|\lambda_{\nu_-}\right|^2\left|\left(\mathbf{c}_{\nu_0}^{\mathrm{in}}\right)_n\right|^2 \tag{S5b}$$

Consequently, we observe that the number of photons is not conserved but rather reduced as the green dashed lines lies below the orange solid line. Importantly, however, we observe that the photon flux associated with the negative-frequency channels is identical to the photon flux of the positive-frequency channels. This shows that the number of pseudophotons is zero for both the incoming and the outgoing field and thus conserved, as we expect for an eigenstate of S_{F} in the broken regime.

In panel (iv), we demonstrate that as the absolute value of the eigenvalue $\left|\lambda_{\nu_-}\right|$ becomes smaller (approaching zero), the corresponding number of photons of the output light field decreases. At the CPA operational condition where $\left|\lambda_{\nu_-}\right| = 0$ (not explicitly shown here), no output light field is produced such that the outgoing photon flux vanishes. Conversely, the number of photons of an eigenvalue $\left|\lambda_{\nu_+}\right|$ is increased during scattering with the extremal case of $\left|\lambda_{\nu_+}\right| = \infty$ corresponding to the scenario of extreme photon amplification of the light field. Finally, also for the state depicted in panel (iv), the photon flux is equally distributed among positive and negative channels. This indicates that this eigenstate has a vanishing pseudophoton content, as is characteristic of eigenstates of S_{F} in the broken symmetry regime.

Finally, we want to clarify the implications of the above discussion for the energy flux of the associated states. A photon-flux normalized state $\mathbf{c}^{\mathrm{in/out}}$ can be transformed into an energy-normalized state $\mathbf{d}^{\mathrm{in/out}}$ via the matrix B as

$$\mathbf{d}^{\mathrm{in/out}} = B\mathbf{c}^{\mathrm{in/out}} \tag{S6}$$

where for the time-varying slab B takes the form

$$\begin{aligned} B = \mathrm{diag}(\ldots, \sqrt{\hbar|\omega_{-1}|}, \sqrt{\hbar|\omega_0|}, \ldots, \\ \ldots, \sqrt{\hbar|\omega_{-1}|}, \sqrt{\hbar|\omega_0|}, \ldots) \end{aligned} \tag{S7}$$

We use this connection for the following observation:

$$\begin{aligned} (\mathbf{d}_\nu^{\mathrm{out}})^\dagger\mathbf{d}_\nu^{\mathrm{out}} &= (\mathbf{c}_\nu^{\mathrm{out}})^\dagger B^\dagger B\mathbf{c}_\nu^{\mathrm{out}} \\ &= |\lambda_\nu|^2(\mathbf{c}_\nu^{\mathrm{in}})^\dagger B^\dagger B\mathbf{c}_\nu^{\mathrm{in}} = |\lambda_\nu|^2(\mathbf{d}_\nu^{\mathrm{in}})^\dagger\mathbf{d}_\nu^{\mathrm{in}} \end{aligned} \tag{S8}$$

This relation tells us that whenever an eigenstate of S_{F} conserves the number of photons (unbroken regime, $\nu = \nu_0$), it also conserves energy, given by $\sum_n \left|\left(\mathbf{d}_\nu^{\mathrm{in}}\right)_n\right|^2 = \sum_n \left|(\mathbf{d}_\nu^{\mathrm{out}})_n\right|^2$. Conversely, a state from the broken

regime ($\nu = \nu_\pm$) neither conserves the number of photons nor its energy content.

Importantly, due the non-orthogonality of the eigenstates of the pseudounitary Floquet scattering matrix, even in the unbroken regime where all eigenstates individually conserve the number of photons and thus their energy content, energy (and the number of photons) is not a conserved quantity for arbitrary states. Superpositions of eigenstates from the unbroken regime will, in general, change their energy (number of photons) upon scattering. On the contrary, the number of pseudophotons is a conserved quantity of any superposition of eigenstates (irrespective if the constituents belong to the unbroken or broken regime).

S3. FAR-FIELD INTENSITY CALCULATIONS FOR THE CPA AND LASING POINTS OF THE TIME-VARYING METASURFACE

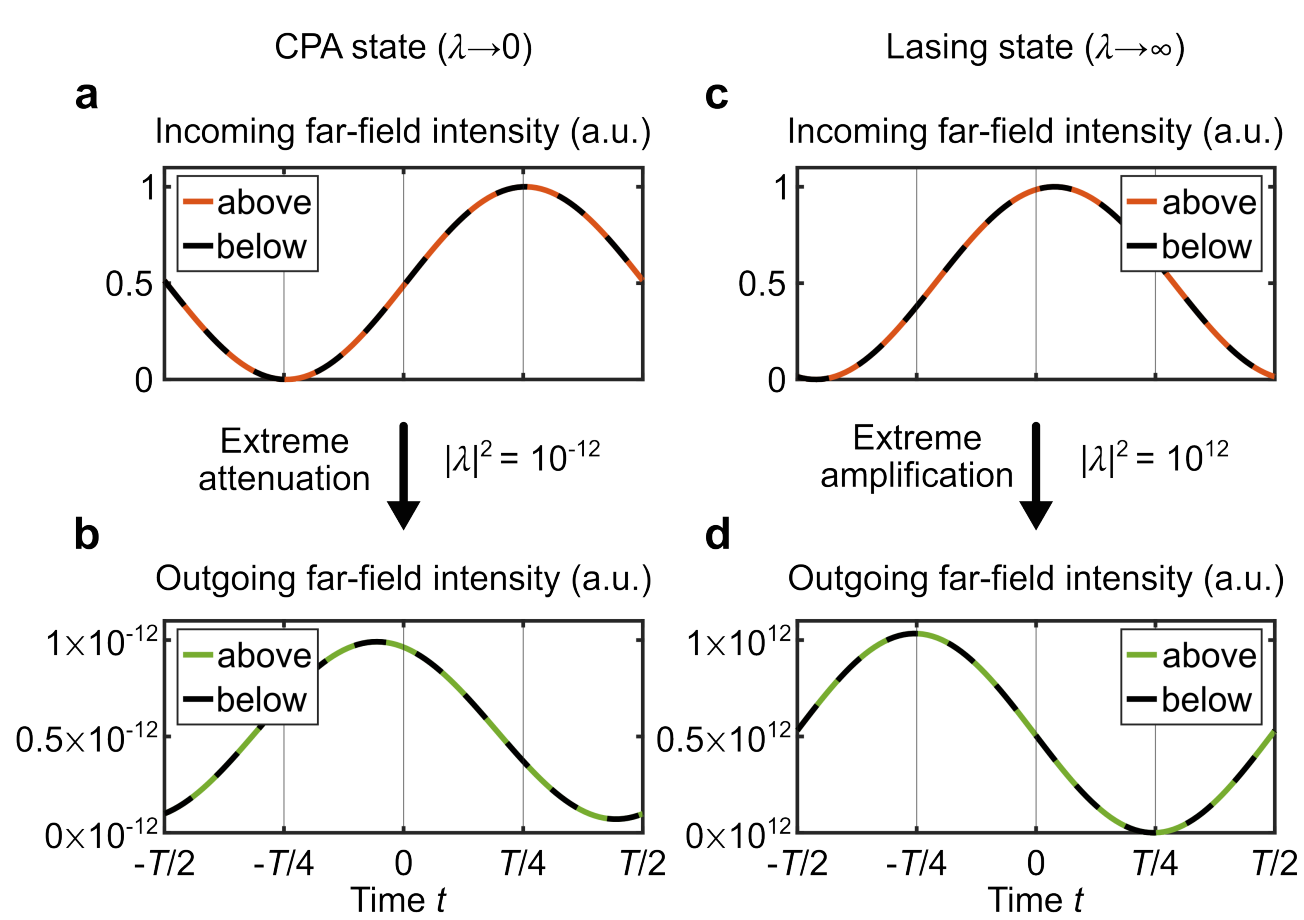

FIG. S2. Space-integrated incoming and outgoing intensities for the CPA and lasing states of the time-varying metasurface (see main text for parameter values) as a function of time t. We evaluate the electric fields in the far-field at $z = \pm 1000 \times Tc$ away from the metasurface and spatially integrate them in the $x-y$ plane. **a** Incoming intensity above the metasurface at $z = 1000 \times Tc$ (red full line) and below the metasurface at $z = -1000 \times Tc$ (black dashed line) and **b** outgoing intensity above (green full line) and below (black dashed line) corresponding the CPA state with an eigenvalue $|\lambda| = 10^{-6}$. An extreme attenuation of the intensity can be observed, and nearly no outgoing field is produced. **c** and **d** Same as **a**, **b** but for the lasing state corresponding to an eigenvalue $|\lambda| = 10^{6}$. Here, we observe an extreme amplification of the incoming light field. We again see the time-reversal symmetry between the incoming CPA and outgoing lasing field.

We plot the temporal intensity distribution in the far-field of the eigenstate corresponding to the minimal eigenvalue (CPA state) in Fig. S2a-b. Specifically, we evaluate the electric field at $z = 1000 \times Tc$ above (full lines) and below (dashed lines) the metasurface in the far-field and spatially integrate the intensity in this $x-y$ plane. The associated eigenvalue is $|\lambda| = 10^{-6}$, resulting in a drastic absorption of the input power such that nearly no outgoing field is produced. Conversely, in Fig. S2c-d, we depict the far-field intensity of the eigenstate to the maximal eigenvalue (lasing state). There, we observe a large amplification of the incoming field corresponding to the eigenvalue $|\lambda| = 10^{6}$ of S_{F}. Again, the time-reversal symmetry between the CPA and the lasing state is apparent (the incoming CPA field is the time-reversed of the outgoing lasing field, and the incoming lasing field is the time-reversed of the outgoing CPA field).

S4. FLOQUET SCATTERING MATRIX OF THE TIME-VARYING SPHERE

We use the Mie theory-based T-matrix method to evaluate S_{F} of the time-varying sphere. We expand the incident field $\mathbf{E}^{\mathrm{inc}}(\mathbf{r},t)$, internal field $\mathbf{E}^{\mathrm{int}}(\mathbf{r},t)$, and scattered field $\mathbf{E}^{\mathrm{sca}}(\mathbf{r},t)$ for the time-varying sphere in a basis of vector spherical waves (VSWs) as

$$\mathbf{E}^{\mathrm{inc}}(\mathbf{r},t) = \sum_{nlms} \tilde{a}^{\mathrm{inc}}_{nlms} \mathbf{F}^{(1)}_{lms}(k_n\mathbf{r}) \mathrm{e}^{-i\omega_n t} \tag{S9a}$$

$$\mathbf{E}^{\mathrm{int}}(\mathbf{r},t) = \sum_{nlms} \left[\sum_i \tilde{a}^{\mathrm{int}}_{ilms} S_{\kappa_i}(\omega_n) \mathbf{F}^{(1)}_{lms}(\kappa_i\mathbf{r}) \right] \mathrm{e}^{-i\omega_n t} \tag{S9b}$$

$$\mathbf{E}^{\mathrm{sca}}(\mathbf{r},t) = \sum_{nlms} \tilde{a}^{\mathrm{sca}}_{nlms} \mathbf{F}^{(3)}_{lms}(k_n\mathbf{r}) \mathrm{e}^{-i\omega_n t} \tag{S9c}$$

where $k_n = \omega_n/c$. Here, $\tilde{a}^{\mathrm{inc}}_{nlms}$, $\tilde{a}^{\mathrm{int}}_{ilms}$, and $\tilde{a}^{\mathrm{sca}}_{nlms}$ represent the incident, internal, and scattered field coefficients, respectively. Furthermore, $\mathbf{F}^{(1)}_{lms}(k_n\mathbf{r})$ [$\mathbf{F}^{(3)}_{lms}(k_n\mathbf{r})$] represent the regular (radiating) VSWs with total angular momentum $l = 1, 2, 3..., l_{\mathrm{max}}$; z-component of angular momentum $m = -l, -l+1, ..., l$; and parity $s = 0, 1$. Here, $s = 0$ represents the transverse-electric (TE), and $s = 1$ represents the transverse-magnetic (TM) mode, respectively. Moreover, l_{max} is the maximum multipolar order used in the expansion. Furthermore, κ_i^2 and $|S_{\kappa_i}\rangle$ represent the eigenvalues and the corresponding eigenvectors, respectively, of the frequency coupling matrix of a time-varying homogeneous medium. The explicit expression of the used frequency coupling matrix is given by [2, Eq. (10)]. Here, $i \in [-N-1, N]$. Note that $|S_{\kappa_i}\rangle$ has $2(N+1)$ entries and $S_{\kappa_i}(\omega_j)$ corresponds to its jth entry.

Next, to find the relation between $|\tilde{a}^{\mathrm{inc}}\rangle$ and $|\tilde{a}^{\mathrm{sca}}\rangle$, we use the following interface conditions on the surface of the sphere [2]

$$\hat{\mathbf{r}} \times \left[\mathbf{E}^{\mathrm{int}}(\mathbf{r},t) - \mathbf{E}^{\mathrm{sca}}(\mathbf{r},t) - \mathbf{E}^{\mathrm{inc}}(\mathbf{r},t) \right] \Big|_{\mathrm{r}=R} = 0 \tag{S10a}$$

$$\hat{\mathbf{r}} \times \left[\mathbf{H}^{\mathrm{int}}(\mathbf{r},t) - \mathbf{H}^{\mathrm{sca}}(\mathbf{r},t) - \mathbf{H}^{\mathrm{inc}}(\mathbf{r},t) \right] \Big|_{\mathrm{r}=R} = 0 \tag{S10b}$$

Here, $\mathbf{H}^{\rm inc}(\mathbf{r},t)$, $\mathbf{H}^{\rm int}(\mathbf{r},t)$, and $\mathbf{H}^{\rm sca}(\mathbf{r},t)$ represent the incident, internal, and scattered magnetic field, respectively. These interface conditions allow us to link the coefficients $|\tilde{a}^{\rm inc}\rangle$ and $|\tilde{a}^{\rm sca}\rangle$ by the Floquet T-matrix $\tilde{T}_0$ as

$$|\tilde{a}^{\rm sca}\rangle = \tilde{T}_0\,|\tilde{a}^{\rm inc}\rangle\ . \tag{S11}$$

Note that $\tilde{T}_0$ is a square matrix with the dimension $2l_{\rm max}(2N)(l_{\rm max}+2)$.

Here, for a given $|\tilde{a}^{\rm sca}\rangle$, the scattered power $P_n^{\rm sca}$ for the frequency ω_n in the far-field is given by [2]

$$P_n^{\rm sca} = \sum_{lms} \frac{c^2\,|\tilde{a}^{\rm sca}_{nlms}|^2}{Z_0\omega_n^2} \tag{S12}$$

Therefore, the photon flux ϕ_n corresponding to $P_n^{\rm sca}$ reads

$$\phi_n = \frac{P_n^{\rm sca}}{\hbar|\omega_n|} = \sum_{lms} \frac{c_0^2\,|\tilde{a}^{\rm sca}_{nlms}|^2}{Z_0\hbar|\omega_n|^3} \tag{S13}$$

where $\hbar$ is the reduced Planck's constant, and Z_0 is the impedance of free space. From Eq. (S13), we note that the scattered field coefficients $|a^{\rm sca}\rangle$ in a photon-flux normalized basis can be obtained by the transformation

$$a^{\rm sca}_{nlms} = \sqrt{\frac{c^2}{Z_0\hbar|\omega_n|^3}}\,\tilde{a}^{\rm sca}_{nlms} \tag{S14}$$

Similarly, for the incident field coefficients $|a^{\rm inc}\rangle$, we can write

$$a^{\rm inc}_{n'l'm's'} = \sqrt{\frac{c^2}{Z_0\hbar|\omega_{n'}|^3}}\,\tilde{a}^{\rm inc}_{n'l'm's'} \tag{S15}$$

Furthermore, combining Eqs. (S11), (S14), and (S15), we get

$$|a^{\rm sca}\rangle = T_0\,|a^{\rm inc}\rangle\ . \tag{S16}$$

Here, T_0 is the Floquet T-matrix of the sphere in a photon-flux normalized basis. The elements of T_0 can be calculated from those of $\tilde{T}_0$ by using the transformation [3]

$$T_0{}^{\{nlms\},\{n'l'm's'\}} = \sqrt{\left|\frac{\omega_{n'}}{\omega_n}\right|^3}\,\tilde{T}_0^{\{nlms\},\{n'l'm's'\}} \tag{S17}$$

Here, the first set of indices enumerates rows, and the second set enumerates columns of the Floquet T-matrices.

Finally, we can compute the Floquet scattering matrix of the time-varying sphere in a photon-flux normalized basis as [4]

$$S_{\rm F} = \mathbb{1} + 2T_0 \tag{S18}$$

Note that the Floquet scattering matrix $S_{\rm F}$ satisfies Eq. (5). Furthermore, $S_{\rm F}$ also satisfies Eq. (11) for permittivity profiles $\epsilon(t)$ that are symmetric with respect to $t=0$.

S5. FLOQUET SCATTERING MATRIX OF THE TIME-VARYING METASURFACE

We expand the incoming field $\mathbf{E}^{\rm in}(\mathbf{r},t)$ and the outgoing field $\mathbf{E}^{\rm out}(\mathbf{r},t)$ from the time-varying metasurface for the Floquet frequency ω and Bloch wavevector $\mathbf{k}_\parallel$ in a basis of plane waves as [5, 6]

$$\mathbf{E}^{\rm in}(\mathbf{r},t) = \sum_{n\mathbf{g}\alpha d} \tilde{u}^{\rm in}_{n\mathbf{g}\alpha d}\,\mathbf{P}_{\mathbf{g}\alpha d}(k_n\mathbf{r})\,\mathrm{e}^{-i\omega_n t} \tag{S19a}$$

$$\mathbf{E}^{\rm out}(\mathbf{r},t) = \sum_{n\mathbf{g}\alpha d} \tilde{u}^{\rm out}_{n\mathbf{g}\alpha d}\,\mathbf{P}_{\mathbf{g}\alpha d}(k_n\mathbf{r})\,\mathrm{e}^{-i\omega_n t} \tag{S19b}$$

where $\mathbf{P}_{\mathbf{g}\alpha d}(k_n\mathbf{r})$ corresponds to a plane wave with the wavevector $\mathbf{k}_{n\mathbf{g}\alpha d} = (\mathbf{k}_\parallel+\mathbf{g}) + (-1)^d\sqrt{k_n^2-(\mathbf{k}_\parallel+\mathbf{g})^2}\,\hat{\mathbf{z}}$, and polarization α. Here, α takes the values 0 and 1 for TE and TM polarized plane waves, respectively. Furthermore, d takes the values 0 and 1 for downward and upward propagating plane waves (with respect to the plane of the metasurface), respectively. Moreover, $\mathbf{g}$ corresponds to a reciprocal lattice vector. In the expansion of the fields $\mathbf{E}^{\rm in}(\mathbf{r},t)$ and $\mathbf{E}^{\rm out}(\mathbf{r},t)$, we assume G_n as the total number of diffraction orders for the frequency ω_n. Here, G_n is chosen such that $|k_n| > |(\mathbf{k}_\parallel+\mathbf{g})|$ for all ω_n. This choice ensures that only the contribution of propagating plane waves is taken into account when evaluating $\mathbf{E}^{\rm in}(\mathbf{r},t)$ and $\mathbf{E}^{\rm out}(\mathbf{r},t)$. Such a choice of G_n is justified as it implies that the sources and the detectors for $\mathbf{E}^{\rm in}(\mathbf{r},t)$ and $\mathbf{E}^{\rm out}(\mathbf{r},t)$ are placed in the far-field of the metasurface.

We connect the plane wave coefficients $|\tilde{u}^{\rm in}\rangle$ and $|\tilde{u}^{\rm out}\rangle$ using the Floquet scattering matrix $\tilde{S}_{\rm F}$ of the metasurface as [5]

$$|\tilde{u}^{\rm out}\rangle = \tilde{S}_{\rm F}\,|\tilde{u}^{\rm in}\rangle\ . \tag{S20}$$

Here, $\tilde{S}_{\rm F}$ is a square matrix with dimension $\sum_{n=-N}^{N-1} 4G_n$.

Note that we numerically evaluate $\tilde{S}_{\rm F}$ using the effective Floquet T-matrix $\tilde{T}_{\rm eff}$ of the metasurface. Using the Floquet T-matrix of an isolated time-varying sphere, $\tilde{T}_{\rm eff}$ can be calculated as

$$\tilde{T}_{\rm eff}(\omega,\mathbf{k}_\parallel) = \left[\mathbb{1} - \tilde{T}_0(\omega)\sum_{\mathbf{R}\neq 0}\tilde{C}^{(3)}(-\mathbf{R})\,\mathrm{e}^{i\mathbf{k}_\parallel\cdot\mathbf{R}}\right]^{-1}\tilde{T}_0(\omega) \tag{S21}$$

Here, $\tilde{C}^{(3)}$ is the translation matrix of VSWs for time-varying metasurfaces (see [5, Eq. (S16)]).

To evaluate $\tilde{S}_{\rm F}$, from $\tilde{T}_{\rm eff}$, we use

$$\tilde{S}_{\rm F} = \mathbb{1} + \tilde{O}\,\tilde{T}_{\rm eff}\,\tilde{I} \tag{S22}$$

Here, $\tilde{O}$ is the coordinate transformation matrix of the scattered field from the spherical wave to the plane wave basis, and $\tilde{I}$ is the coordinate transformation matrix of

the incident field from the plane wave to the spherical wave basis [5, Eqs. (S8), (S13)].

Next, we write the power flux P_n^{out} carried by the outgoing fields $\mathbf{E}^{\text{out}}(\mathbf{r}, t)$ for the frequency ω_n in the far-field as [5]

$$P_{\text{out}}^{n} = \frac{c}{2Z_0} \sum_{\mathbf{g}\alpha d} \frac{|k_{z,n\mathbf{g}\alpha d}|}{|\omega_n|} |\tilde{u}^{\text{out}}_{n\mathbf{g}\alpha d}|^2 \tag{S23}$$

Here, $k_{z,n\mathbf{g}\alpha d}$ refers to the z-component of the wavevector $\mathbf{k}_{n\mathbf{g}\alpha d}$. The photon flux ϕ_n corresponding to P_n^{out} is given by

$$\phi_n = \frac{c}{2Z_0} \sum_{\mathbf{g}\alpha d} \frac{|k_{z,n\mathbf{g}\alpha d}|}{\hbar\omega_n^2} |\tilde{u}^{\text{out}}_{n\mathbf{g}\alpha d}|^2 \tag{S24}$$

From Eq. (S24), we note that the outgoing field coefficient $|u^{\text{out}}\rangle$ in a photon-flux normalized basis can be obtained by the transformation

$$u^{\text{out}}_{n\mathbf{g}\alpha d} = \sqrt{\frac{c|k_{z,n\mathbf{g}\alpha d}|}{2Z_0\hbar\omega_n^2}}\,\tilde{u}^{\text{out}}_{n\mathbf{g}\alpha d} \tag{S25}$$

Similarly, for the incoming field coefficients $|u^{\text{in}}\rangle$, we can write

$$u^{\text{in}}_{n'\mathbf{g}'\alpha' d'} = \sqrt{\frac{c|k_{z,n'\mathbf{g}'\alpha' d'}|}{2Z_0\hbar(\omega_n')^2}}\,\tilde{u}^{\text{in}}_{n'\mathbf{g}'\alpha' d'} \tag{S26}$$

Furthermore, combining Eqs. (S20), (S25), and (S26), we get

$$|u^{\text{out}}\rangle = S_{\text{F}}\,|u^{\text{in}}\rangle\ . \tag{S27}$$

Here, S_{F} is the Floquet scattering matrix of the metasurface in a photon flux normalized basis. The elements of S_{F} can be calculated from those of $\tilde{S}_{\text{F}}$ using the transformation

$$S_{\text{F}}^{\{n\mathbf{g}\alpha d\},\{n'\mathbf{g}'\alpha' d'\}} = \left|\frac{\omega_{n'}}{\omega_n}\right| \sqrt{\left|\frac{k_{z,n\mathbf{g}\alpha d}}{k_{z,n'\mathbf{g}'\alpha' d'}}\right|} \times \tilde{S}_{\text{F}}^{\{n\mathbf{g}\alpha d\},\{n'\mathbf{g}'\alpha' d'\}} \tag{S28}$$

We arrange $|u^{\text{out}}\rangle$ and $|u^{\text{in}}\rangle$ following the conventions: $|u^{\text{out}}\rangle = (\mathbf{u}^{\text{out}}_{\downarrow}, \mathbf{u}^{\text{out}}_{\uparrow})^{\text{T}}$ and $|u^{\text{in}}\rangle = (\mathbf{u}^{\text{in}}_{\uparrow}, \mathbf{u}^{\text{in}}_{\downarrow})^{\text{T}}$, respectively. Here, $\mathbf{u}^{\text{out}}_{\uparrow}$ ($\mathbf{u}^{\text{in}}_{\uparrow}$) correspond to the upward propagating outgoing (incoming) and $\mathbf{u}^{\text{out}}_{\downarrow}$ ($\mathbf{u}^{\text{in}}_{\downarrow}$) correspond to the downward propagating outgoing (incoming) field coefficients. Importantly, we arrange the entries of the S_{F} matrix as,

$$S_{\text{F}} = \begin{pmatrix} S_{\downarrow\uparrow} & S_{\downarrow\downarrow} \\ S_{\uparrow\uparrow} & S_{\uparrow\downarrow} \end{pmatrix} \tag{S29}$$

which is crucial for correctly describing the spectral locations of the EPs as shown in [7]. Note that S_{F} satisfies Eq. (5). Furthermore, S_{F} also satisfies Eq. (11) for permittivity profiles $\epsilon(t)$ that are symmetric with respect to $t = 0$.

[1] X. Wang, P. Garg, M. S. Mirmoosa, A. G. Lamprianidis, C. Rockstuhl, and V. S. Asadchy, Expanding momentum bandgaps in photonic time crystals through resonances, Nat. Photonics **19**, 149 (2025).

[2] G. Ptitcyn, A. Lamprianidis, T. Karamanos, V. Asadchy, R. Alaee, M. Müller, M. Albooyeh, M. S. Mirmoosa, S. Fan, S. Tretyakov, and C. Rockstuhl, Floquet–Mie theory for time-varying dispersive spheres, Laser Photonics Rev. **17**, 2100683 (2023).

[3] A. G. Lamprianidis, *Generalized transition matrix methods for the analysis of linear nanophotonic systems*, Ph.D. thesis, Karlsruher Institut für Technologie (KIT) (2024).

[4] P. Waterman, T-matrix methods in acoustic scattering, J. Acoust. Soc. Am. **125**, 42 (2009).

[5] P. Garg, A. G. Lamprianidis, D. Beutel, T. Karamanos, B. Verfürth, and C. Rockstuhl, Modeling four-dimensional metamaterials: a T-matrix approach to describe time-varying metasurfaces, Opt. Express **30**, 45832 (2022).

[6] D. Beutel, A. Groner, C. Rockstuhl, and I. Fernandez-Corbaton, Efficient simulation of biperiodic, layered structures based on the T-matrix method, J. Opt. Soc. Am. B **38**, 1782 (2021).

[7] A. Novitsky, D. Lyakhov, D. Michels, A. A. Pavlov, A. S. Shalin, and D. V. Novitsky, Unambiguous scattering matrix for non-Hermitian systems, Phys. Rev. A **101**, 043834 (2020).
